# Specific Antibodies Induced by Immunization with Hepatitis B Virus-Like Particles Carrying Hepatitis C Virus Envelope Glycoprotein 2 Epitopes Show Differential Neutralization Efficiency

**DOI:** 10.3390/vaccines8020294

**Published:** 2020-06-10

**Authors:** Anna Czarnota, Anna Offersgaard, Anne Finne Pihl, Jannick Prentoe, Jens Bukh, Judith Margarete Gottwein, Krystyna Bieńkowska-Szewczyk, Katarzyna Grzyb

**Affiliations:** 1 Laboratory of Virus Molecular Biology, Intercollegiate Faculty of Biotechnology, University of Gdańsk, 80-309 Gdańsk, Poland; anna.czarnota@biotech.ug.edu.pl (A.C.); krystyna.bienkowska-szewczyk@biotech.ug.edu.pl (K.B.-S.); 2 Copenhagen Hepatitis C Program (CO-HEP), Department of Infectious Diseases, Hvidovre Hospital, 2650 Hvidovre, Denmark; anna.offersgaard@regionh.dk (A.O.); anne.finne.pihl@regionh.dk (A.F.P.); jprentoe@sund.ku.dk (J.P.); jbukh@sund.ku.dk (J.B.); jgottwein@sund.ku.dk (J.M.G.); 3 Department of Immunology and Microbiology, Faculty of Health and Medical Sciences, University of Copenhagen, 2200 Copenhagen, Denmark

**Keywords:** hepatitis C virus, vaccine, virus like particles (VLPs), HBV small surface antigen (sHBsAg)

## Abstract

Hepatitis C virus (HCV) infection with associated chronic liver diseases is a major health problem worldwide. Here, we designed hepatitis B virus (HBV) small surface antigen (sHBsAg) virus-like particles (VLPs) presenting different epitopes derived from the HCV E2 glycoprotein (residues 412–425, 434–446, 502–520, and 523–535 of isolate H77C). Epitopes were selected based on their amino acid sequence conservation and were previously reported as targets of HCV neutralizing antibodies. Chimeric VLPs obtained in the *Leishmania tarentolae* expression system, in combination with the adjuvant Addavax, were used to immunize mice. Although all VLPs induced strong humoral responses, only antibodies directed against HCV 412–425 and 523–535 epitopes were able to react with the native E1E2 glycoprotein complexes of different HCV genotypes in ELISA. Neutralization assays against genotype 1–6 cell culture infectious HCV (HCVcc), revealed that only VLPs carrying the 412–425 epitope induced efficient HCV cross-neutralizing antibodies, but with isolate specific variations in efficacy that could not necessarily be explained by differences in epitope sequences. In contrast, antibodies targeting 434–446, 502–520, and 523–535 epitopes were not neutralizing HCVcc, highlighting the importance of conformational antibodies for efficient virus neutralization. Thus, 412–425 remains the most promising linear E2 epitope for further bivalent, rationally designed vaccine research.

## 1. Introduction

Hepatitis C virus (HCV) poses a serious medical problem as the number of new infections continues to be on the rise. Thus, there are at least 2 million acute infections annually, and in 80% of these individuals chronic hepatitis will develop [1]. Over time this has resulted in at least 70 million people chronically infected with HCV worldwide with a significant risk for progression to liver cirrhosis and hepatocellular carcinoma [1]. In recent years, new antiviral therapies based on direct-acting antivirals (DAAs) have been devised [2]. However, due to poor diagnostic coverage and high treatment cost, only a minor fraction of HCV-infected individuals will receive DAA therapy. In addition, emerging resistance to DAAs is expected to compromise treatment efficacy [3,4]. Thus, development of an effective prophylactic vaccine against HCV is required to control this deadly virus.

While both neutralizing antibodies and T cells contribute to spontaneous clearance of HCV infection [5,6], most antiviral vaccines protect by neutralizing antibodies induced by whole virus or virus-like particle (VLP) antigens [7,8]. For HCV, neutralizing antibodies induced by a subunit envelope protein vaccine had protective effects in chimpanzees [9], while a viral vector-based vaccine inducing T cells did not protect against chronic HCV infection in chimpanzees and humans [10,11]. Thus, development of an HCV vaccine inducing antibodies is a current research focus. 

The high genetic heterogeneity of HCV poses an obstacle for development of an anti-HCV vaccine. At present, we can distinguish six major HCV genotypes with proven epidemiological relevance, while more recently genotypes 7 and 8 have been reported in a few individuals in central Africa and India, respectively [12,13]. Further, there are at least 90 HCV subtypes [14]. Genotypes 1, 2, and 3 are distributed across the globe with genotype 1 being the most common worldwide. Among genotype 1 isolates, subtypes 1a and 1b are most prevalent with predominance of subtype 1b. Genotype 4 is most common in the Middle East and Central and North Africa, genotype 5 in South Africa, and genotype 6 in Southeast Asia [12,13,15,16].

As the prime target of host neutralizing antibodies, the HCV envelope glycoproteins E1 and E2 located on the surface of the viral particle show extensive genetic heterogeneity, in particular in the hypervariable region 1 (HVR1) of E2. E1 and E2 form a covalent heterodimer embedded in the lipid envelope of the virus particle and play a crucial role in the infection process by interacting with the HCV-entry receptors, including tetraspanin CD81 [17]. In view of the high variability of E1E2, an effective vaccine should preferentially raise neutralizing antibodies against genetically conserved regions of the E1E2 heterodimer to elicit antibodies able to neutralize different HCV strains [18,19].

A number of relatively conserved epitopes able to induce cross-neutralizing antibodies have been identified, including two well-defined CD81-binding regions on E2; residues 412–423 (epitope I) (all E2 positions are assigned using H77C polyprotein numbering) [18,20,21], previously proposed as the target for anti-HCV therapeutic antibodies, and residues 434–446 (epitope II) [22,23]. Downstream of the epitope II sequence there are additional neutralizing epitopes, spanning residues 502–520 and residues 523–535 (epitope III), which also play a key role in HCV cell entry by influencing interactions between the viral particle and the CD81 receptor [24,25,26,27]. These regions offer interesting targets for rational vaccine design.

Although synthetic peptides can trigger immune responses, peptide vaccines are generally weakly immunogenic and require carriers for delivery [28,29]. Recently, there has been an increased interest in VLPs as platforms for immunogen exposition. VLP-based vaccines have several advantages over conventional immunogens. First, due to their virus-like appearance and highly organized structure, VLPs are effective as immunostimulants as they are able to elicit both humoral and cellular responses. Second, they can easily be produced in heterologous expression systems to large quantities and can easily be purified. Finally, VLPs do not contain virus genetic material and are considered safe in comparison to replicating vectors [30].

One of the best-characterized VLP-forming proteins is the hepatitis B virus (HBV) small surface antigen (sHBsAg). Yeast-derived sHBsAg forms particles of 22 nm in diameter and is currently used worldwide as the commercial recombinant hepatitis B virus vaccine. The sHBsAg tertiary structure features a highly conserved, hydrophilic loop containing major B-cell epitopes, also known as the “a”-determinant [31,32]. Because of its immunogenic potential, sHBsAg has been applied as an antigen carrier to deliver foreign sequences to induce humoral and cellular immune responses [33,34,35,36,37,38]. At the outset of this study, proof-of-concept was obtained that sHBsAg particles could be used as carriers of small HCV epitopes inserted into the antigenic external hydrophilic loop to induce strong and specific antibody responses [38,39,40].

In the present study, we designed a panel of sHBsAg VLPs with epitopes derived from the HCV E2 glycoprotein inserted into the “a”-determinant. The E2 epitopes were selected due to their relative sequence conservation and since they were reported to be targets of HCV neutralizing antibodies. The particles were produced in a non-conventional, cost-efficient *Leishmania tarentolae* expression system, purified by ultracentrifugation, and then used to immunize mice. This study is a follow-up of our previously published work in which we described the expression of chimeric sHBsAg particles carrying the highly conserved HCV 412–425 epitope and characterized binding of vaccine-induced antibodies to denatured HCV genotype 1–6 E2 glycoproteins [39]. In this study, we widened the panel of tested HCV E2 epitopes and evaluated the ability of vaccine-induced antibodies to bind native HCV genotype 1–6 E1E2 complexes and their potential to cross-neutralize cell culture infectious HCV (HCVcc) of genotype 1–6.

## 2. Materials and Methods

### 2.1. Plasmids

**Figure 1:**
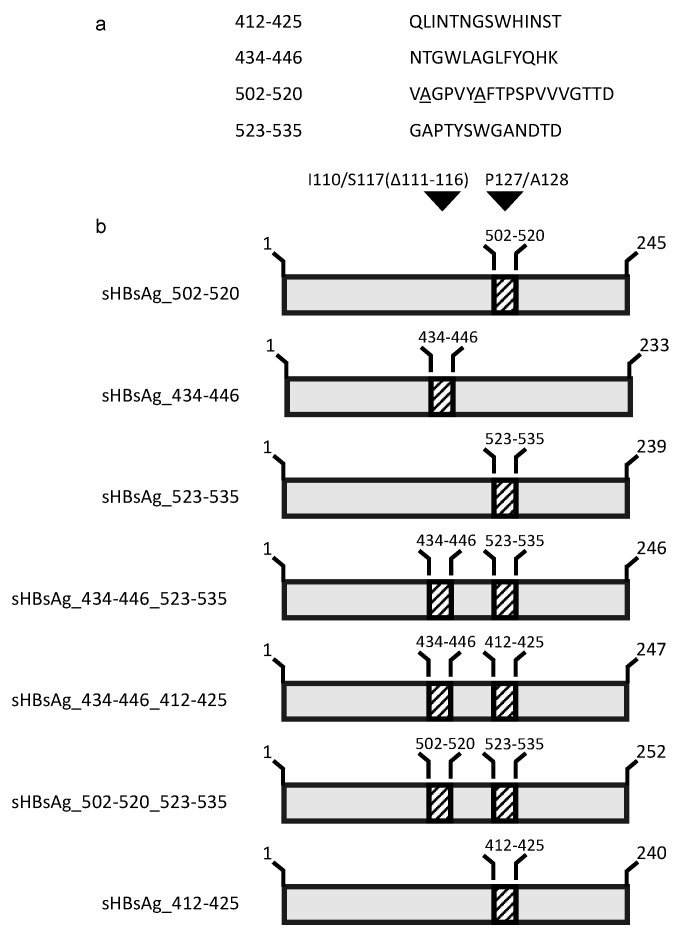
Construction of chimeric proteins. (**a**) Epitope sequences were derived from the hepatitis C virus (HCV) isolate H77C (GenBank accession no. AF011751). The underlined alanine corresponds to the substituted cysteine. (**b**) Recombinant constructs were generated by insertion of the sequences coding for HCV E2 epitopes into the sequence coding for the hydrophilic loop of the hepatitis B virus (HBV) small surface antigen (sHBsAg) protein at positions I110/S117(Δ111–116) and/or P127/A128—insertion sites are marked with black arrows.

Figure 1 summarizes the construction of the chimeric genes for HBV/HCV particle generation. The regions of the HCV E2 glycoprotein expressing residues 412–425, 434–446, 502–520, and 523–535 (isolate H77C, GenBank accession no. AF011751 [41]) were inserted in the HBV subtype adw2 sHBsAg (GenBank accession no. AF397207.1). Insertions of the HCV E2 epitopes into the hydrophilic loop of the sHBsAg protein were performed at position I110/S117 (Δ111–116)—for construct sHBsAg_434–446; at position P127/A128—for constructs sHBsAg_502–520 and sHBsAg_523–535; or at both positions—for constructs sHBsAg_434–446_523–535, sHBsAg_434–446_412–425, and sHBsAg_502–520_523–535. Additionally, constructs carrying epitope 502–520 had the cysteine residues at positions 503 and 508 substituted with alanine (C503A and C508A). Construction of the chimeric gene coding for construct sHBsAg_412–425 has been described earlier [39].

The constructs were obtained by gene synthesis using *L. tarentolae*-adapted codons (Thermo Scientific, Waltham, MA, USA). Synthetized genes were ligated into the BglII–NotI restriction sites in the pLEXSY_I-blecherry3 vector (Jena Bioscience, Jena, Germany).

For expression of HCV E2 glycoprotein in human epithelial kidney (HEK) 293 cells for ELISA, we used plasmids coding for full-length E1E2, derived from HCV genotypes (isolates): 1a(H77C) [41]; 1b(12.6); 2a(2.4); 2b(1.1); 3a(1.9); 4a(21.16); 5a(14.4); and 6a(5.8) [42].

### 2.2. Leishmania tarentolae Cultivation and Protein Expression

The chimeric sHBsAg-based proteins were expressed using the inducible LEXSY expression system in accordance with the manufacturer’s instructions (Jena Bioscience). Briefly, the plasmids were transfected into *L. tarentolae* cells by electroporation. The transfected cells were selected with bleomycin (100 µg/mL) in suspension culture. Subsequently, recombinant cell lines were cultivated in 25 cm^2^ tissue culture flasks filled with 10 mL of selective medium supplemented with hemin protected from light at 26 °C. The T7 promoter driven transcription was induced by adding tetracycline to the final concentration of 15 µg/mL. The cells were grown in agitated culture, in 500 mL shake flasks for 72 h at 26 °C, aiming at a final optical density of 4–5 at 600 nm (OD_600_).

### 2.3. SDS-PAGE and Western Blot

An analysis of the particle expression was carried out by SDS-PAGE of *Leishmania tarentolae* cell lysates using 4–12% gradient Bis–Tris gels in MES SDS running buffer. After electrophoresis, proteins were transferred onto a PVDF membrane by electroblotting, and subsequently the membranes were blocked overnight at 4 °C with 3% nonfat milk in TBST [TBS buffer, 0.1% (*v*/*v*) Tween-20]. Following blocking, the membranes were incubated for 1 h at room temperature (RT) with primary anti-HBsAg antibodies diluted in 3% nonfat milk in TBST, washed with TBST, and then incubated with goat anti-rabbit secondary horseradish peroxidase-conjugated antibodies (Santa Cruz Biotechnology, Dallas, TX, USA). The results were obtained through development in the substrate for enhanced chemiluminescence (Thermo Scientific).

### 2.4. Cell Lysis and Ultracentrifugation

One hundred milliliters of tetracycline-induced cell culture were centrifuged at 4 °C, 8800× *g* for 15 min. The cell pellet was immediately resuspended in 10 mL of ice-cold lysis buffer [PBS buffer, 0.6% (*v*/*v*) Tween-20]. The cells were sonicated, and the suspension clarified by centrifugation at 4 °C, 8000 rpm, for 35 min; the supernatant was left for 16–24 h at RT to form particles. Subsequently, the lysate was layered on an OptiPrep (Sigma-Aldrich, Saint Louis, MO, USA) gradient formed in ultra-clear tubes [2 mL of 30% (*v*/*v*) OptiPrep, 2 mL 24% (*v*/*v*) OptiPrep, 1.5 mL 18% (*v*/*v*) OptiPrep, 1.5 mL 12% (*v*/*v*) OptiPrep, and 1.5 mL 6% (*v*/*v*) OptiPrep in PBS] and ultracentrifuged at 90,000× *g* for 16 h at 4 °C. Then, 500 µL fractions were harvested and analyzed by a western blot using anti-HBsAg rabbit polyclonal antibodies (OriGene, Rockville, MD, USA). Fraction purity was analyzed by SDS-PAGE with Coomassie R-250 staining. The fractions with the highest number of particles were pooled, and protein concentration was measured by Bradford assay. Finally, the OptiPrep solution was replaced with PBS using Amicon Ultra 100 K centrifugal filter units (Merck Millipore, Burlington, MA, USA). Additionally, samples containing sHBsAg_434–446 particles were normalized against sHBsAg_412–425 protein using anti-HBsAg rabbit polyclonal antibodies (OriGene). These samples were used for further analysis and immunization.

### 2.5. Electron Microscopy

For visualization of the particles, the OptiPrep gradient fractions were diluted 1:5 in PBS and deposited on carbon-coated 200 mesh nickel grids. Negative staining was performed using 2% uranyl acetate. Following the staining, the samples were analyzed using a transmission electron microscope (University of Gdańsk, Gdańsk, Poland).

### 2.6. Immunization Protocol

Groups of 6 female BALB/c mice, 6–8 weeks of age, were immunized subcutaneously with squalene-based oil-in-water nanoemulsion adjuvant (Addavax, InvivoGen). The mice were immunized with 15 µg of protein on day 0, and with 10 µg on days 14 and 28. The mice used as negative controls were immunized with PBS-adjuvant mixture alone. All experiments on animals were conducted by an accredited company (Tri-City Academic Laboratory Animal Centre, Medical University of Gdańsk, Gdańsk, Poland), in accordance with the current guidelines for animal experimentation. The protocols were approved by the Local Committee on the Ethics of Animal Experiments of the University of Science and Technology in Bydgoszcz (Permit Number: 38/2018). All procedures were performed under isoflurane anesthesia, and all effort was taken to minimize suffering.

### 2.7. Analysis of the Antibody Response by ELISA

Mouse sera were collected two weeks after the last immunization and pooled according to experimental groups. The antibody response against HCV E2 epitopes was measured by direct solid-phase ELISA. Pre-blocked, streptavidin-coated plates (Thermo Scientific) were incubated with biotinylated peptides at 5 µg/mL (JPT-Innovative Peptide Solutions, Berlin, Germany) for 16 h at 4 °C. Following the coating and washing, serially diluted mouse sera were added to the wells and incubated for 1 h at RT. Goat anti-mouse secondary HRP-conjugated antibodies (Santa Cruz Biotechnology, Dallas, USA) were used for detection. Similarly, the antibody response against sHBsAg protein was tested using ELISA plates coated with *Pichia pastoris*-derived sHBsAg protein at 5 µg/mL (OriGene, Rockville, MD, USA). The plates were then blocked for 2 h with 3% (*w*/*v*) BSA in PBST [PBS buffer, 0.05% (*v*/*v*) Tween-20], and serially diluted mouse sera were added to the wells as described above.

For testing serum cross-reactivity to E1E2 complexes, HEK293 cells were transfected with plasmids expressing glycoproteins E1E2 derived from different HCV genotypes. The cells were washed with PBS buffer and lysed in lysis buffer (PBS buffer, 0.5% Triton X-100) 72 h after transfection. The clarified cell lysates were normalized against each other using anti-E2 goat polyclonal antibodies (Bio-Rad, Hercules, CA, USA) and later used to perform ELISA in denatured/reduced and native conditions. For the denatured ELISA, the HEK293 cell lysates were first diluted 10× in PBS buffer containing 50 mM DTT and 2% SDS and incubated for 10 min at 100 °C; for the native ELISA, the HEK293 cell lysates were diluted 10× in PBS alone. Next, the cell lysates were moved onto ELISA plates precoated with *Galanthus nivalis* lectin (GNA) and incubated for 16 h at 4 °C. Following that, the plates were blocked in PBST with 3% BSA and used immediately or stored at −20 °C. The pooled mouse sera were tested in 1:1000 PSB dilutions containing 0.3% BSA. Finally, the binding of the antibodies to the recombinant proteins was detected by goat anti-mouse HRP-conjugated secondary antibodies diluted to 1:2500 (Santa Cruz Biotechnology, Dallas, TX, USA) and the 3,3′,5,5′-Tetramethylbenzidine substrate.

### 2.8. IgG Purification from Mouse Sera

IgGs were isolated from mouse serum using NAb Protein G Spin Kit (Thermo Scientific, Waltham, MA, USA) in accordance with the manufacturer’s instructions. Briefly, 300 µL of pooled mouse sera was transferred into the spin columns and incubated for 30 min at RT. Subsequently, purified IgGs were eluted from the resin with low pH elution buffer and concentrated using Amicon Ultra 100 K centrifugal filter units (Merck Millipore, Burlington, MA, USA).

### 2.9. Virus Stocks for HCVcc Neutralization Assay

HCVcc virus stocks with sequence confirmed E1 and E2 were produced in Huh7.5 cells in DMEM supplemented with 10% fetal bovine serum (FBS) and penicillin/streptomycin (P/S) as previously described [43]. As HCVcc, JFH1-based HCV recombinants with Core-NS2 of the following genotypes (isolates) and specified cell culture adaptive substitutions were used: 1a(TN) with R1408W [44], 1b(J4) with F886L and Q1496L [43], 2a(J6) [45], 2b(J8) [43], 3a(S52) with I793S and K1404Q [43], 4a(ED43) with T827A and T977S [46], 5a(SA13) with A1022G and K1119R [47], and 6a(HK6a) with F350S and N417T [43].

### 2.10. HCVcc Neutralization Assay

The cell-based in vitro HCV neutralization assay was done as previously described [47] with modifications. Briefly, 7 × 10^4^ Huh7.5 cells were plated per well in a poly-D-lysine-coated 96-well plate (Nunc, Roskilde, Denmark) in DMEM supplemented with FBS and P/S. The next day, a 96-well pre-plate with virus and antibody mixes was prepared. The amount of virus stock yielding counts of 20–100 focus forming units (FFU)/well in pilot assays was diluted in medium to a total volume of 7 µL and mixed with 3 µL serially diluted purified IgG; for virus-only wells, 3 µL medium was added. Virus/antibody mixes were incubated at 37 °C and 5% CO_2_ for 1.5 h. Subsequently, medium was added to a total volume of 40 µL per well and the solution was transferred to the cell plate. After 4.5 h incubation at 37 °C and 5% CO_2_, cells were washed with PBS and incubated with fresh medium. Each antibody concentration was tested in triplicates. At least 6 virus-only wells and negative control wells without virus and antibody mixes were included in each assay. At 48 h post-infection, cells were fixed with methanol and incubated with PBS containing 0.5% (*w*/*v*) BSA and 0.1% (*w*/*v*) skimmed milk for 1 h and subsequently incubated with AffiniPure Fab Fragment Goat Anti-Mouse IgG (H+L) (Jackson Immuno Research, West Grove, USA) diluted in PBS at 100 µg/mL for 1 h and immunohistochemically stained. Antibodies were diluted in PBS containing 0.5% (*w*/*v*) BSA and 0.1% (*w*/*v*) skimmed milk; the primary antibody 9E10^42^ was diluted 1:5000, and the secondary antibody ECL anti-mouse IgG HRP-linked whole antibody (GE Healthcare, Amersham, UK) was diluted 1:500; both were incubated overnight at 4 °C and visualized with Pierce™ DAB Substrate Kit (Thermo Scientific, Waltham, MA, USA). FFU were counted and neutralization was evaluated as described [48]. Briefly, an ImmunoSpot series 5 UV analyzer (CTL Europe GmbH, Bonn, Germany) and customized software were used for automated counting of FFU. Percentage of neutralization was calculated by relating the number of FFU in each well to the mean number of FFU in virus-only wells.

## 3. Results

### 3.1. Expression of the Chimeric sHBsAg-Based Particles

The sequences coding for HCV (genotype 1a, isolate H77C [41]) E2 glycoprotein regions spanning residues 412–425, 434–446, 502–520, and 523–535 were inserted individually or in combinations into the sequence of the major antigenic loop of HBV sHBsAg protein at positions corresponding with amino acids I110/S117 (Δ111–116) and P127/A128 (Figure 1a,b), previously reported to support peptide insertions without an impact on particle formation. Additionally, cysteine residue substitution (C→A) in epitope 502–520 was performed in order to minimize the risk of tertiary conformation disturbance.

**Figure 2:**
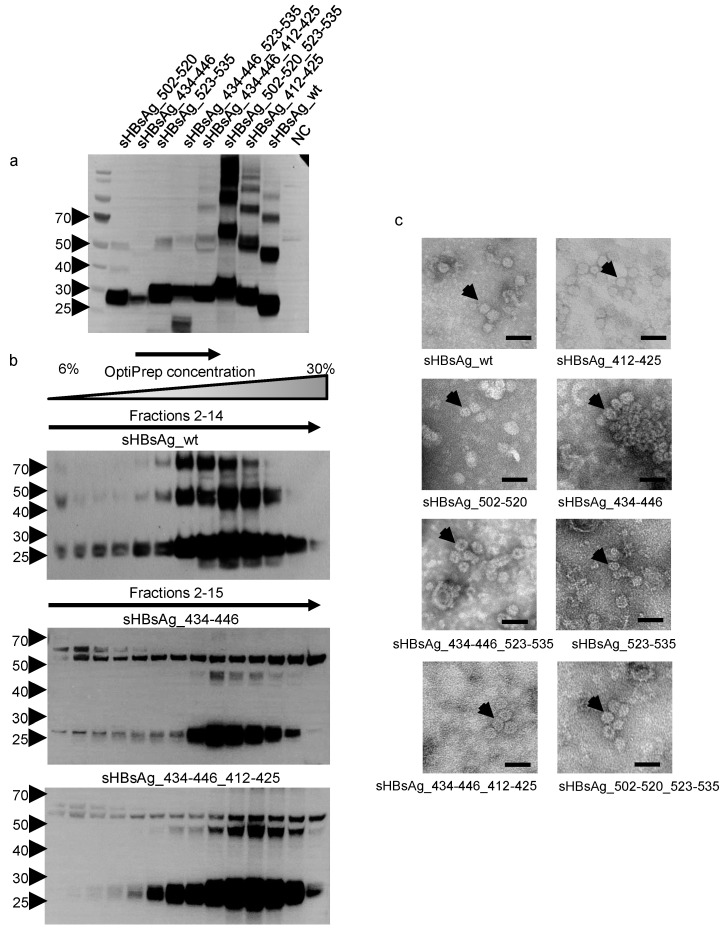
Expression and characterization of the chimeric proteins. (**a**) Western blot analysis of the chimeric proteins expressed in *L. tarentolae*. Cell lysates were separated using SDS-PAGE and detected with the anti-HBsAg antibody. The lysate from wild-type *L. tarentolae* cells was used as the negative control (NC). Bands of higher molecular mass correspond with the multimeric forms of the proteins. On the left protein ladder, the molecular weight in kDa is given. (**b**) In order to concentrate and partially purify the chimeric particles, lysates from the *L. tarentolae* cell cultures expressing chimeric proteins were placed on top of an OptiPrep density gradient. Seventeen fractions of 0.5 mL were harvested from top to bottom. The aliquots of fractions 2–15 were then analyzed using a western blot with anti-HBsAg antibodies. On the left protein ladder, the molecular weight in kDa is given. (**c**) Electron micrographs of chimeric sHBsAg-based particles. After concentration on the Optiprep density gradient, the chimeric particles were stained with uranyl acetate and analyzed using electron microscopy. Observed particles were approximately 20–30 nm in diameter. Black arrows: Chimeric virus-like particles (VLPs). Scale bar: 50 nm.

Chimeric VLPs were produced in high-density cell cultures using the tetracycline-inducible *Leishmania tarentolae* expression system. The expression of proteins was confirmed by SDS-PAGE of cell lysates, followed by a western blot with sHBsAg-specific antibodies (Figure 2a). In reducing conditions, the molecular masses of monomers of the chimeric proteins fell in the range of 27–35 kDa. The difference in the molecular masses of the monomers was probably associated not only with the insertion of the HCV E2-derived epitopes, but also with the molecular mass of the additional N-glycans. In comparison to the wild-type sHBsAg, chimeric particles carrying the 412–425 epitope contain two additional glycosylation sites at positions N417 and N423. The chimeric particles exposing the 523–535 epitope contain one additional glycosylation site at position N532. For all chimeric proteins, multimers of higher molecular mass were also detected. Notably, during cultivation of the recombinant *Leishmania tarentolae* cells, we consistently observed a lower protein level for sHBsAg_434–446 in comparison to those of other chimeric proteins and the sHBsAg wild-type (wt) control. This finding is in agreement with the poor growth kinetics indicated by relatively low OD_600_ of the sHBsAg_434–446 *Leishmania tarentolae* culture reached after 72h of induction, in comparison to the other recombinant cell cultures (Table 1).

The chimeric VLPs were concentrated and partially purified from the cell lysate by ultracentrifugation on an OptiPrep gradient. Most of the sHBsAg protein monomer was distributed in fractions 9–13 (Figure 2b). The figure represents an example of the sHBsAg protein distribution in the OptiPrep gradient for sHBsAg_wt and two chimeric VLPs: sHBsAg_434–446 and sHBsAg_434–446_412–425. All other chimeric VLPs demonstrated analogous sHBsAg distribution profiles after gradient ultracentrifugation (Figure S1). Particle assembly was confirmed by transmission electron microscopy. The sHBsAg-positive fractions showed spherical particles approximately 20–30 nm in diameter (Figure 2c). In summary, although some differences in the particle expression level were observed, we were able to confirm sHBsAg-based chimeric protein production and particle formation for all designed VLPs.

### 3.2. Immunogenicity of the Chimeric sHBsAg-Based Particles

**Figure 3:**
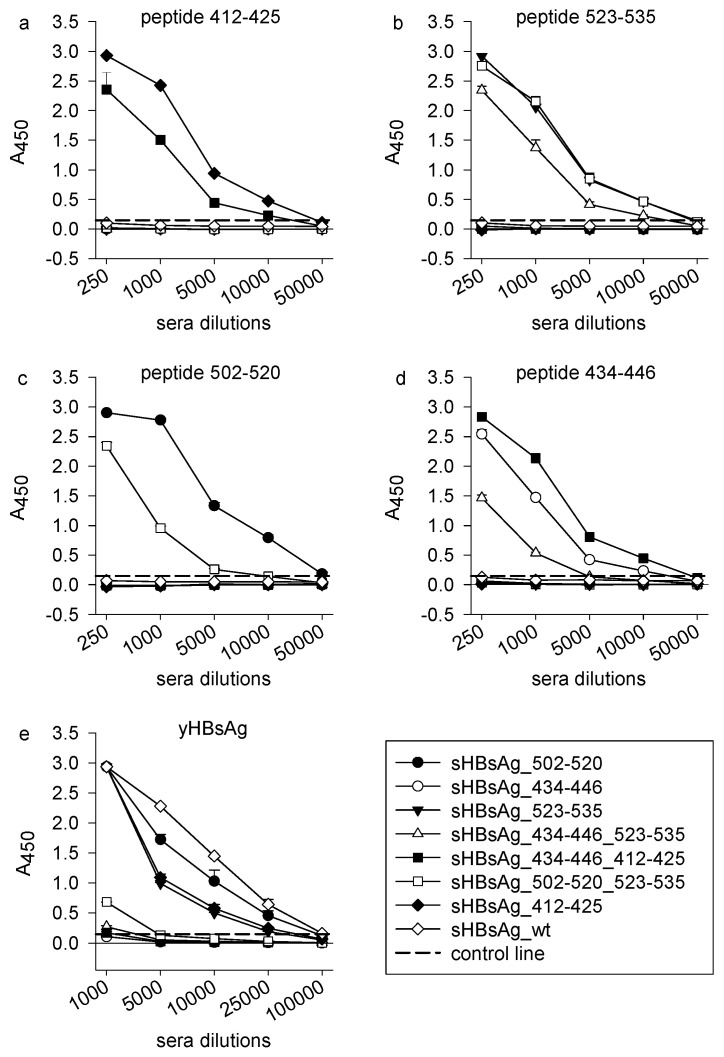
Immunization with chimeric sHBsAg-based particles elicited specific antibody responses in mice. (**a**–**d**) For analysis of binding of immune sera to HCV peptides, streptavidin-coated microplates were coated with 5 µg/mL of biotinylated synthetic peptides covering epitopes 412–425, 523–535, 502–520, and 434–446 of HCV E2. (**e**) For analysis of binding of immune sera to yeast-derived sHBsAg (yHBsAg), ELISA plates were coated with 5 µg/mL of purified sHBsAg protein derived from *Pichia pastoris*. The dilution factors of the mouse sera are shown on axis *x*. The mean A_450_ values are shown on axis *y*. The background signal from the negative control mouse sera was subtracted from the obtained results. The data represent the results from two independent experiments performed in duplicate, and error bars indicate standard deviations. The dashed horizontal line represents the cutoff value (three times the mean background value).

To investigate immunogenicity, eight groups of BALB/c mice were immunized subcutaneously with fractions containing different VLPs. All mice were immunized using the squalene-based Addavax adjuvant, an MF59 analogue, the latter being licensed in Europe for human use. After mouse immunizations, we found strong and specific antibody responses against biotinylated peptides, corresponding with the E2 sequence exposed on the surface of the chimeric VLPs. Antibody titers approaching 5 × 10^4^ were observed for sera from all mouse groups (Figure 3a–d). The results evidenced that immunization with particles carrying single and double foreign epitopes could elicit a strong and specific antibody response against all exposed HCV epitopes.

A detailed serum characterization showed strong cross-reactivity to purified yeast-derived sHBsAg protein (yHBsAg) for the sera collected from the mice immunized with the sHBsAg_502–520, sHBsAg_523–535, and sHBsAg_412–425 VLPs (Figure 3e), with the antibody endpoint titer comparable to that of wild-type sHBsAg VLPs. Weaker or no responses were observed following immunization using sHBsAg_434–446 and all VLPs exposing two epitopes (sHBsAg_434–446_523–535, sHBsAg_434–446_412–425, and sHBsAg_502–520_523–535).

**Figure 4:**
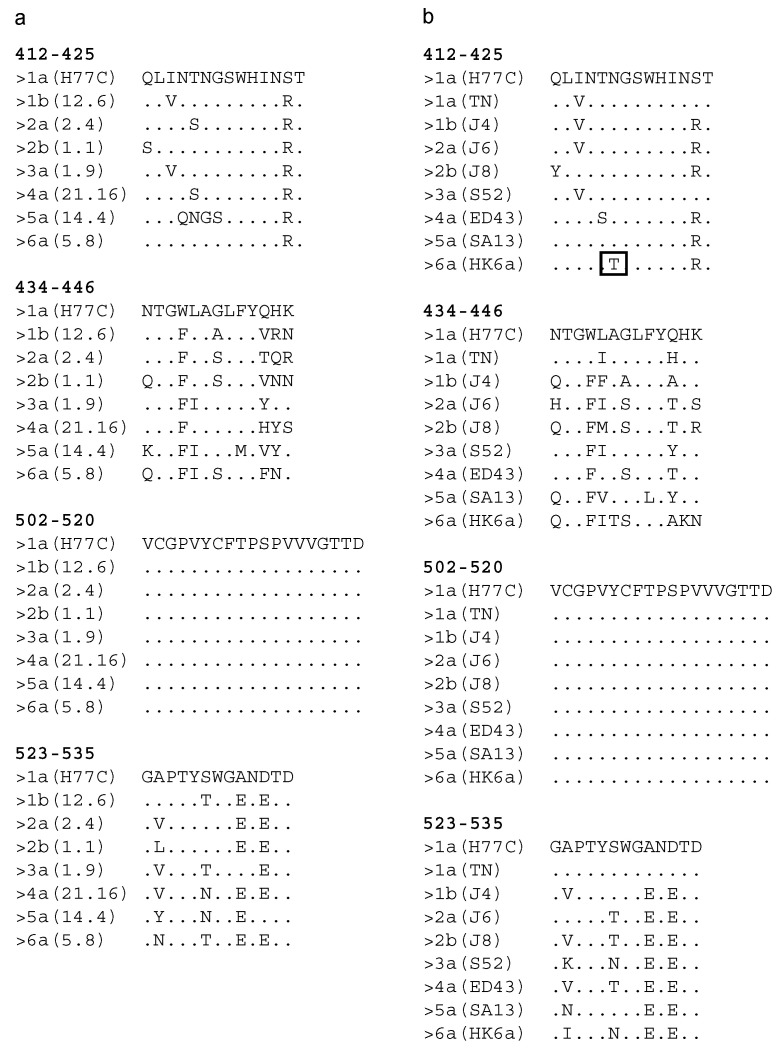
Amino acid alignment showing the sequence conservation of the regions 412–425, 434–446, 502–520, and 523–535 for full-length E1E2 (**a**) and HCVcc isolates (**b**) used in this study. The sequence derived from the HCV genotype 1a isolate H77C (GenBank accession number AF011751) is used as the reference and indicated on the top of each alignment. Other sequences are designated according to genotype (isolate). Amino acids conserved in reference to the H77C sequence are marked with dots. For 6a (HK6a), the boxed threonine is a cell culture adaptive substitution.

We next tested cross-reactivity of the sera from the immunized mice against the E1E2 complexes of HCV genotypes 1–6 in ELISA assays. Under reduced/denatured conditions (Table 2a), six out of seven antisera recognized E1E2 from HCV genotypes 1a, 1b, 2a, 2b, 3a, 4a, and 6a. The immune sera derived from the mice immunized with particles carrying epitope 412–425 only failed to recognize the 5a (14.4) E1E2. This is in line with previous results, as the 5a (14.4) isolate shows five amino acid changes in region 412–425 in comparison to the source sequence derived from the H77C isolate (Figure 4a) [39]. The antibodies elicited by VLPs carrying the 502–520 or 523–535 epitopes were able to bind all tested E1E2. Finally, sHBsAg_434–446 VLPs did not elicit any antibodies capable of binding to HCV E1E2. We then carried out ELISA under native conditions (Table 2b). Interestingly, we observed significant variation among the antisera in the binding profile to the native E1E2 complexes expressed in mammalian cells. The broadest cross-reactivity was found for sera from mice immunized with VLPs carrying the 412–425 epitope (sHBsAg_412–425 and sHBsAg__434–446_412–425), as they only failed to recognize the non-conserved 5a (14.4) E1E2. Antisera from mice immunized with VLPs carrying the 523–535 epitope (sHBsAg_523–535, sHBsAg_434–446_523–535, and sHBsAg_502–520_523–535) recognized E1E2 from genotypes 1a, 1b, 2a, and 5a, but not 3a and 4a. Additionally, a limited recognition of E1E2 of genotypes 2b and 6a was observed. At last, sera from mice immunized with sHBsAg_502–520 and sHBsAg_434–446 VLPs failed to recognize native E1E2 from any tested genotype.

**Figure 5:**
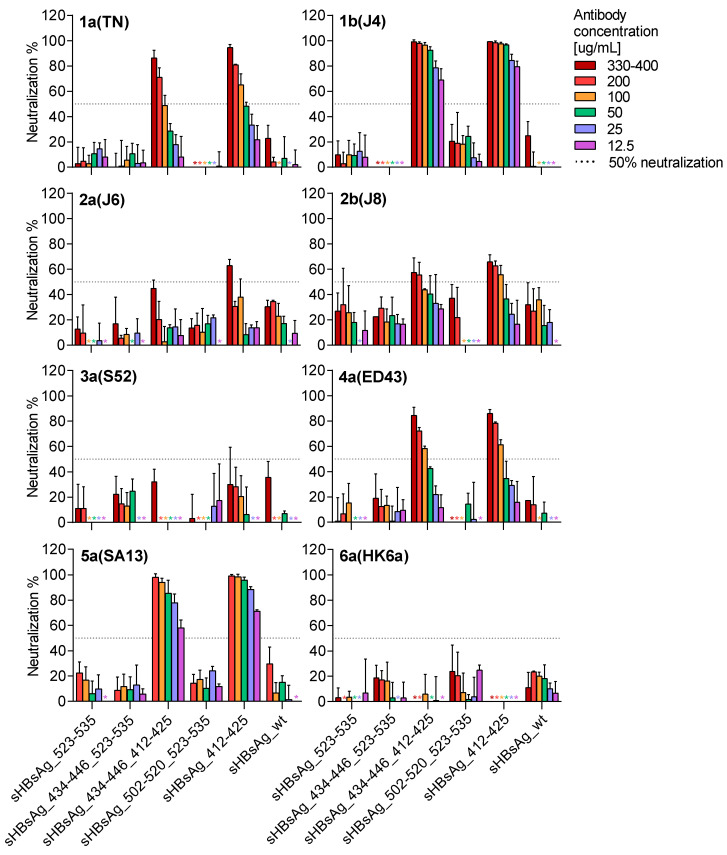
Chimeric VLP-induced antibodies cross-neutralized different HCV genotypes. Viruses were incubated with IgGs purified from the sera of immunized mice and pooled for each experimental group. The antibodies were pre-incubated with viruses of HCV genotypes (isolates) 1a (TN), 1b (J4), 2a (J6), 2b (J8), 3a (S52), 4a (ED43), 5a (SA13), and 6a (HK6a) for 1.5 h, followed by incubation of Huh7.5 cells with antibody-virus mixes for 4.5 h. The neutralization effect (%) was determined relative to cultures infected with the respective viruses in the absence of antibodies. The data represent the mean values from three technical replicates and error bars indicate standard deviations. The dashed horizontal line marks 50% virus neutralization. Neutralization values of ≤0% are not shown; *, asterisks are added in cases where error bars and neutralization values are <0%.

Finally, we evaluated the neutralizing potency of IgG purified from the mouse sera against genotypes 1–6 HCVcc. As shown in Figure 5, a neutralizing effect was only observed for antibodies derived from the mice immunized with VLPs carrying the 412–425 epitope (sHBsAg_412–425 and sHBsAg_434–446_412–425). These antibodies showed the most potent neutralization efficacy against genotype 1a, 1b, 4a, and 5a viruses, with 80–100% neutralization at the highest IgG concentrations. In addition, they were able to neutralize genotype 2a and 2b viruses with lower efficacy, approaching ~60% neutralization at the highest IgG concentrations. In contrast, for genotype 3a and 6a viruses, less than 50% neutralization was observed at the highest concentrations tested. For genotype 6a viruses, this is likely explained by the essential cell culture adaptive substitution N417T in the 412–425 region (Figure 4b) [43].

Thus, immunogenicity studies revealed that all designed VLPs were immunogenic, as they were eliciting antibodies recognizing specifically the HCV E2 epitope used for immunization. However, only sera from mice immunized with VLPs carrying epitopes 412–425 and 523–535 were able to recognize native E1E2 heterodimers and only VLPs with epitope 412–425 were capable of eliciting a strong HCV cross-neutralizing antibody response.

## 4. Discussion

We developed a panel of sHBsAg-based VLPs carrying moderately to highly conserved and immunogenic epitopes derived from the HCV E2 glycoprotein using the economically attractive *Leishmania tarentolae* expression system. Following immunization with the adjuvant Addavax, which is similar to the MF59 adjuvant licensed for human use in Europe, VLPs carrying the 412–425 epitope elicited potent cross-neutralizing IgGs with isolate specific neutralization differences, suggesting differences in epitope accessibility on HCVcc.

Previously, chimeric sHBsAg-based VLPs were developed carrying the HCV E2 HVR1 [38,40]. This approach might be problematic due to the high variability of the HVR1 protein sequence. Considering the high genetic diversity and structural flexibility of HCV E2, efforts should be made to target immune responses to conserved epitopes. Therefore, we here explored several HCV E2 epitopes as potential vaccine antigens.

Epitope I (residues 412–423) is an important target for broadly neutralizing antibodies and has been proposed as the target of antibody-based therapy [18,20,21]. Our results highlight the ability of epitope I to induce HCV cross-reactive antibodies. In previous research, we found sera of sHBsAg_412–425-immunized mice to bind E2 glycoproteins derived from different HCV genotypes under denaturing conditions by western blotting [39]. Here, we demonstrated that sera induced by immunization with VLPs carrying epitope I recognized E1E2 complexes derived from different genotypes under native conditions by ELISA, suggesting that epitope I is accessible in the native E1E2 complexes. Most importantly, purified IgG targeting epitope I showed an impressive HCV cross-neutralization potency.

While genotype 2a and 2b viruses showed comparatively low neutralization sensitivity, we observed highly efficient neutralization of genotype 1a, 1b, 4a, and 5a viruses. It should be noted that although the antibodies directed against the 412–425 epitope failed to recognize 5a (14.4) E1E2 in the native ELISA, they effectively neutralized 5a (SA13) HCVcc only differing at one residue in the 412–425 epitope from H77C. Observed neutralization efficacies were comparable to those reported for efficient human monoclonal antibodies [49]. In contrast, antibodies to epitope I failed to neutralize genotype 3a and 6a viruses. For the 6a virus, the lack of neutralization sensitivity can most probably be explained by the presence of the “glycan shift” substitution N417T, which is critical for cell culture adaptation, also of another genotype 6a isolate [50]. It was previously described that a single substitution N417S or N417T resulted in a shift of the N-glycosylation site from N417 to N415 and effectively prevented recognition of epitope I by some antibodies [20,51,52]. Future neutralization studies with genotype 6a HCVpp not depending on this cell culture adaptive substitution could prove whether genotype 6a isolates with native epitope I sequence are neutralized by sHBsAg_412–425-induced antibodies.

Interestingly, our data suggest that epitope I might be shielded in the studied genotype 3a virus and to a lesser extend in the genotype 2a and 2b viruses, as the efficiently neutralized 1a virus had the same epitope I sequence as the 3a virus, and the efficiently neutralized 1b virus had the same epitope I sequence as the 2a virus. This hypothesis is supported by the finding that AP33 Fab fragments, targeting epitope I, neutralized the 1b virus used in this study with high efficacy and the studied 2a and 2b viruses with lower efficacy, while no significant neutralization of the used 3a virus was observed (Prentoe, personal communication). It has previously been reported that HCV envelope features, such as HVR1-mediated epitope protection, determined differences in neutralization susceptibility rather than just sequence diversity [53]. Future research possibly involving structural analysis will be required to prove the hypothesis on differential shielding of epitope I in different HCV isolates. Moreover, in future research, it would be interesting to investigate if these observed effects are genotype or isolate specific, by developing and/or using already developed HCVcc, preferably without cell culture adaptive substitutions in the envelope proteins.

We investigated immunogenicity of three additional relatively conserved epitopes localized in the E2 CD81-binding region comprising residues 434–446, 502–520, and 523–535. Each of these epitopes has previously been described as important for binding of both neutralizing and non-neutralizing anti-HCV antibodies. However, most of these antibodies were described to be conformation-sensitive and recognized discontinuous epitopes located within the CD81 binding region [22,26,54,55]. Similarly, we found that these epitopes were immunogenic and that antibodies targeting these epitopes recognized synthetic peptides and, except for antibodies targeting epitope 434–446, also denatured E1E2. In contrast, antibodies targeting these epitopes were less efficient in recognizing E1E2 in native ELISA and were not neutralizing HCVcc. This suggests that residues 434–446, 502–520, and 523–535 might not be accessible in the E1E2 complex and on HCVcc and thus, induction of antibodies targeting conformational epitopes covering these regions might be required for efficient virus neutralization. The role of antibodies binding residues 434–446 (epitope II) is still under debate. It was reported that such antibodies enhanced HCV neutralization mediated by antibodies targeting epitope I in a synergistic manner [56,57]. On the other hand, it was also suggested that epitope II might be associated with non-neutralizing antibodies mediating antibody-interference by hindering binding of neutralizing antibodies targeting epitope I. Thus, depletion of epitope II-specific antibodies from the serum samples collected from HCV-positive patients [58,59] as well as E1E2 immunized chimpanzees [59] and humans [60] improved neutralizing activity of antibodies targeting epitope I [58,59,60].

Residues 523–535 (epitope III) localize to the C-terminal part of the CD81 binding loop and are an important target for human conformation-sensitive, broadly neutralizing antibodies [61]. Even though antibodies induced by VLPs carrying epitope 523–535 were binding native E1E2 of several isolates, they failed to neutralize HCVcc. Thus, also according to prior evidence, antibodies targeting conformational but not linear epitopes within this region might be required for efficient neutralization [62]. Moreover, the 523–535 region is suggested to adopt different conformations [63,64], which might influence epitope accessibility.

The less well characterized residues 502–520 were proposed as a possible fusion peptide [65], and, more recently, to be of importance for interaction with HCV entry receptors [26]. Antibodies induced by VLPs carrying residues 502–520 were binding denatured but not native E1E2 of all tested isolates and failed to neutralize HCVcc. This could be explained by poor accessibility of the 502–520 epitope, as it is located within the E2 β-sandwich structure [25,26]. In line with this hypothesis, antibodies targeting this region were detected after animal immunizations using a subunit vaccine, but were not induced during natural infection of chimpanzees and humans [24]. It is also possible that C503A and C508A substitutions in the 502–520 epitope (Figure 1) hindered the binding of the sHBsAg_502–520 VLP-elicited antibodies to the native E1E2 heterodimer.

The obtained results proved that exposition of a single epitope on the sHBsAg particle does not necessarily disturb the ability of chimeric particles to induce an immune response against the carrier protein. Thus, sera from mice immunized with most VLPs exposing a single epitope yielded titers of anti-sHBsAg antibodies that were comparable to that of the sera from mice immunized with the wild-type sHBsAg VLPs. This phenomenon is consistent with previously published data [34,38,39,40]. In contrast, insertion of the 434–446 epitope reduced the particle’s ability to elicit sHBsAg-specific antibodies. This might be caused by changes in the carrier protein conformation or overall particle formation. This hypothesis is supported by our observation that sHBsAg proteins including HCV residues 434–446 at position P127/A128 were unable to assemble into VLPs (unpublished data). Even though this hindrance was overcome by insertion at position I110/S117 (Δ111–116), growth of *Leishmania tarentolae* cells expressing sHBsAg__434–446 and expression of these VLPs was significantly reduced compared to the other VLPs (Figure 2a, Table 1). In addition, inclusion of two HCV epitopes into the hydrophilic loop of sHBsAg interfered strongly with the antibody response elicited against the carrier protein. Thus, if an sHBsAg-based VLP vaccine should feature different HCV epitopes and protect against both HCV and HBV, a mixture of VLPs carrying single HCV epitopes might be more suitable than exposure of different epitopes on the surface of a single VLP.

A recent study by Wei et al. [66] also explored immunogenicity of sHBsAg-based VLPs carrying different HCV epitopes (residues 313–327 in E1 as well as 412–423, 523–535, and HVR1 in E2). Less than 50% neutralization of genotype 1a, 1b, and 2a HCVcc in comparison to pre-immunization control serum was observed for serum from mice immunized with single VLPs. The fact that Wei et al. observed limited neutralization with antibodies induced by epitope III might be explained by different presentation of the 523–535 epitope on the surface of the VLPs, due to the insertion of a HIS-tag. However, direct comparison of our results with the study by Wei et al. is hindered due to the lack of specification of the dilutions of mouse sera used for HCVcc neutralization and use of serum instead of purified IgG in neutralization assays, which might be problematic, as interfering serum components might perturb assay results [67].

In this study, we have shown that sHBsAg-based VLPs are able to successfully present different linear HCV epitopes. Elicited antibodies specifically target linear epitopes within the E2 glycoprotein and could in the future be used in studies addressing epitope accessibility for different HCV isolates. Among all tested VLPs, only those comprising 412–425 or 523–535 epitopes were able to induce antibodies recognizing native E1E2 complexes. An HCV neutralization effect was only observed for antibodies induced by VLPs carrying the 412–425 epitope. Although epitope I is suggested to be hidden from the humoral immune response in HCV-infected individuals [68], chimeric particles carrying this epitope elicited high titers of cross-reactive and cross-neutralizing antibodies against HCV while preserving anti-sHBsAg immunity. Interestingly, isolate-specific differences in neutralization efficacy of the antibodies elicited using epitope I suggested isolate-specific epitope accessibility. Future in vivo studies could potentially verify the effectiveness of vaccine-induced immunity; however, at present there are no robust immunocompetent animal models permissive for HCV [15]. In light of the data from the present study, region 412–425 derived from the HCV E2 glycoprotein remains the most promising epitope for further sHBsAg-based bivalent prophylactic vaccine research. In conclusion, vaccination with sHBsAg_412–425 VLPs in combination with additional antigen capable of inducing conformation-dependent antibodies would be beneficial for successful development of a pan-genotypic vaccine against HCV.

## Figures and Tables

**Table 1:** OD_600_ of *Leishmania tarentolae* cell cultures. The measurement was performed for *L. tarentolae* recombinant cell lines expressing chimeric proteins 72 h after tetracycline induction. The data represent the mean values from a minimum of three independent procedures.

	OD_600_
sHBsAg_502–520	4.9
sHBsAg_434–446	2.4
sHBsAg_523–535	4.8
sHBsAg_434–446_523–535	4.8
sHBsAg_434–446_412–425	4.5
sHBsAg_502–520_523–535	5.0
sHBsAg_412–425	5.3
sHBsAg_wt	4.9

**Table 2:** Cross-reactivity of immune sera to E1E2 complexes from different HCV genotypes in (**a**) reduced/denatured and (**b**) native conditions. The sera dilution factor was 1:1000. “+” sections suggest signal of high intensity (A_450_ > 0.5 for native conditions; A_450_ > 1.5 for reduced/denatured conditions), indicating strong binding. “−“ sections suggest low intensity (A_450_ < 0.15), indicating no appreciable binding. “+/−“ sections suggest moderate intensity (0.15 ≤ A_450_ ≤ 0.5 for native conditions), indicating moderate binding. The background signal from the sHBsAg wt serum was subtracted from the obtained results. The data represent the mean values from two independent experiments performed in duplicate.

**(a)**								
**Reduced/Denatured**	**Genotype (Isolate)**							
MOUSE GROUP	1a (H77C)	1b (12.6)	2a (2.4)	2b (1.1)	3a (1.9)	4a (21.16)	5a (14.4)	6a (5.8)
sHBsAg_502–520	+	+	+	+	+	+	+	+
sHBsAg_434–446	-	-	-	-	-	-	-	-
sHBsAg_523–535	+	+	+	+	+	+	+	+
sHBsAg_434–446_523–535	+	+	+	+	+	+	+	+
sHBsAg_434–446_412–425	+	+	+	+	+	+	-	+
sHBsAg_502–520_523–535	+	+	+	+	+	+	+	+
sHBsAg_412–425	+	+	+	+	+	+	-	+
**(b)**								
**Native**	**Genotype (Isolate)**							
MOUSE GROUP	1a (H77C)	1b (12.6)	2a (2.4)	2b (1.1)	3a (1.9)	4a (21.16)	5a (14.4)	6a (5.8)
sHBsAg_502–520	-	-	-	-	-	-	-	-
sHBsAg_434–446	-	-	-	-	-	-	-	-
sHBsAg_523–535	+	+	+	+/−	-	-	+	+/−
sHBsAg_434–446_523–535	+	-	+	+/−	+/−	-	+	+/−
sHBsAg_434–446_412–425	+	+	+	+	+/−	+	-	+/−
sHBsAg_502–520_523–535	+	+	+	+	-	-	+	-
sHBsAg_412–425	+	+	+	+	+	+	-	+

## Supplementary Materials

The following are available online at https://www.mdpi.com/2076-393X/8/2/294/s1, Figure S1: OptiPrep density gradient lysates from the *Leishmania tarentolae* cell cultures expressing chimeric proteins.
