# Supplementary material for: Specific Antibodies Induced by Immunization with Hepatitis B Virus-Like Particles Carrying Hepatitis C Virus Envelope Glycoprotein 2 Epitopes Show Differential Neutralization Efficiency
Source: Vaccines (Basel). 2020 Jun 10;8(2):294. doi: 10.3390/vaccines8020294 (PMC7350033; doi:10.3390/vaccines8020294)
Supplement: Supplementary file 1 [file vaccines-08-00294-s001.pdf]

vaccines

MDPI

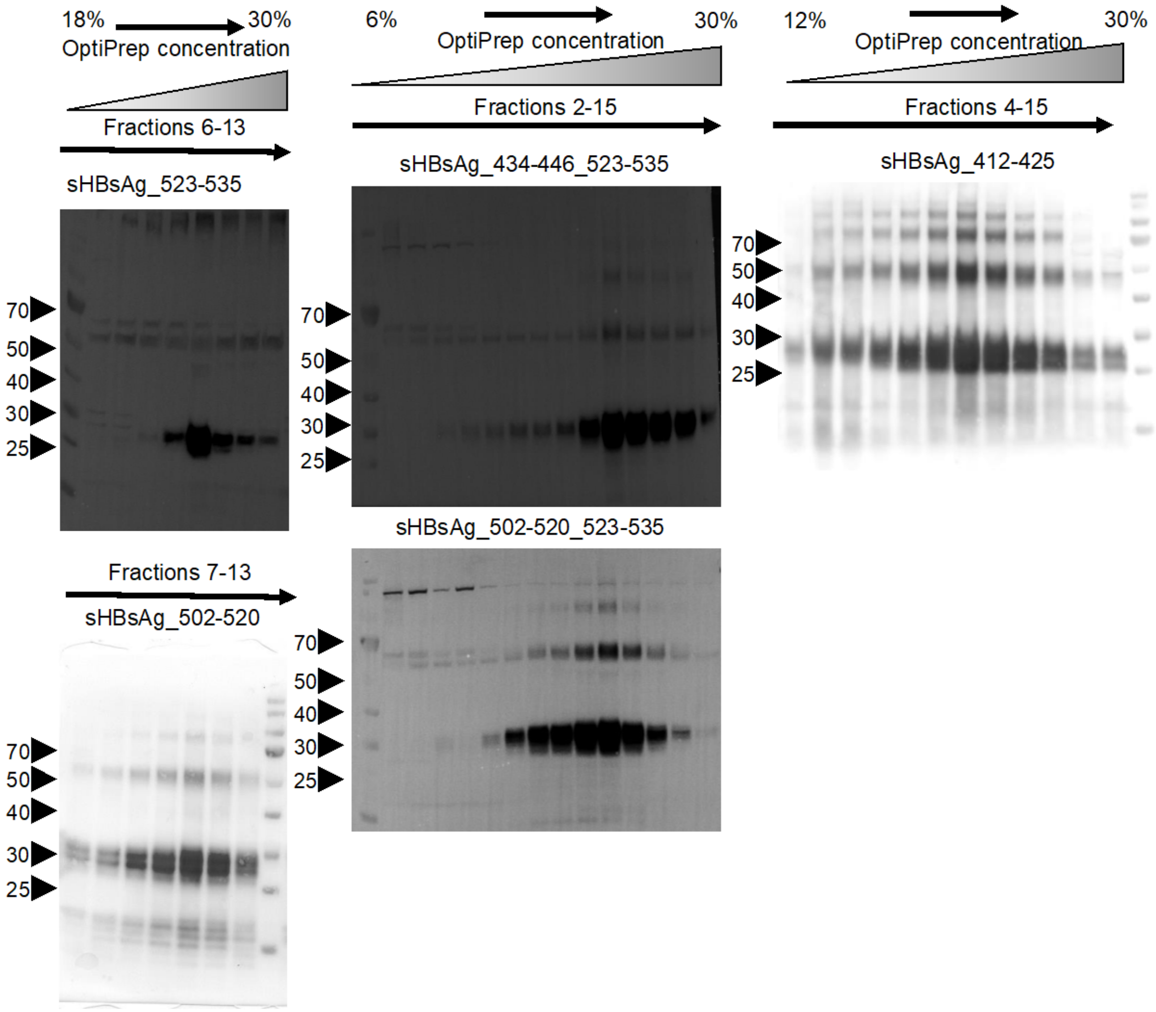

Figure S1. OptiPrep density gradient lysates from the *L. tarentolae* cell cultures expressing chimeric proteins. Seventeen fractions of 0.5 mL were harvested from top to bottom. The aliquots were then analyzed using western blot with anti-HBsAg antibodies. On the left, protein ladder, the molecular weight in kDa is given.
